# Managing chronic widespread pain in primary care: a qualitative study of patient perspectives and implications for treatment delivery

**DOI:** 10.1186/s12891-016-1194-5

**Published:** 2016-08-22

**Authors:** Penny Bee, John McBeth, Gary J. MacFarlane, Karina Lovell

**Affiliations:** 1 School of Nursing, Midwifery & Social work, University of Manchester, Manchester, UK; 2 Arthritis Research UK Centre for Epidemiology, University of Manchester, Manchester, UK; 3 Centre for Musculoskeletal Research, University of Manchester, Manchester, UK; 4 School of Medicine, Medical Sciences and Nutrition, University of Aberdeen, Aberdeen, UK; 5 Aberdeen Centre for Arthritis and Musculoskeletal Health, University of Aberdeen, Aberdeen, UK

**Keywords:** Chronic widespread pain, Treatment acceptability, Patient perspectives, Qualitative

## Abstract

**Background:**

Clinical guidelines recommend a combination of physical, pharmacological and psychological treatments for chronic widespread pain, but published accounts of treatment acceptability are lacking.

**Methods:**

Semi-structured interviews (*n* = 44) nested within a randomised controlled trial comparing the clinical and cost effectiveness of prescribed exercise, cognitive behavioural therapy (CBT), and combined exercise and CBT to treatment as usual for adults with chronic widespread pain.

**Results:**

Three main themes emerged from the data: i) the illness context (how people experience chronic pain and associated health services); ii) the identity context (how people react to their symptoms and accommodate these within themselves) and iii) the intervention context (the extent and manner by which the trial interventions models aligned with these responses).

Referral to a prescribed exercise programme resonated most closely with participants’ tendency to attribute pain to a structural or mechanical defect. Psychological therapy brought with it connotations of social judgement, deviance and stigma. Experience of psychological therapy often exceeded expectation. Participants who engaged in cognitive reflection and behavioural adaptation reported an upward identity shift independent of increased physical exercise behaviour.

**Conclusions:**

A logical rationale for a health intervention is in itself insufficient to ensure uptake and participation. Potential differences in treatment meaning emphasise the importance of acknowledging different phases of illness acceptance and of providing the most appropriate treatment option for the stage of reconciliation. Health service providers must not only understand people’s own perceptions of chronic widespread pain but also the broader spheres of influence in which this pain is experienced.

## Background

Chronic widespread pain has an estimated population prevalence of 11–15 % [1–3] and the associated personal and societal burdens are high. CWP is a disorder characterised by diffuse body pain that has persisted for at least three months. It is the cardinal feature of fibromyalgia syndrome, a disorder diagnosed when CWP co-occurs with a high number of tender points [4].

Chronic widespread pain is associated with lost work productivity [5], mental ill health [6] and reduced quality of life [7]. UK data suggest that newly diagnosed patients receive almost double the number of primary care visits and prescribed medicines to those without the condition [8]. In the US, mean per-patient costs in the first 6 months following diagnosis are estimated at $3481; costs that include pain and non–pain-related medications, medical tests and procedures and emergency department consultations [9].

The development of clinically-effective and cost-effective pain management strategies for chronic widespread pain is challenged by syndrome complexity and equivocal causal mechanisms. International, evidence-based guidelines recommend a combination of physical, pharmacological and psychological treatments [10, 11]; although the relative contribution of each and the magnitude of gain that may be conferred through a multi-component approach remain ill-defined. Systematic reviews [12–14] suggest that physical exercise and cognitive behavioral therapy (CBT) both hold promise as discrete treatment options.

Current models of chronic pain propose that the somatic symptoms arise from a complex interplay of biological, sociological and psychological determinants [15]. The relative roles that these different variables may play in mediating pain symptoms have thus been the focus of much research attention. Uncertainty regarding the diagnostic underpinnings of CWP has precipitated a range of studies examining the influence of affective responses and/or self-efficacy for pain control on functional capacity and quality of life [16]. These studies provide valuable insights into potential mediators of chronic pain, but are unable to shed light on patients’ treatment preferences or intervention experience. Published accounts of patients perspectives on treatment acceptability are lacking.

This paper reports the findings of a qualitative study nested within a large, randomised controlled trial. The MUSICIAN trial, which allocated 442 primary care participants to one of four arms, compared the clinical and cost effectiveness of three psychosocial interventions (Prescribed exercise, CBT and exercise-CBT combined) with treatment as usual for chronic pain. Trial results have been published previously [17].

This qualitative study, conducted with a sub-sample of trial participants aimed to explore participants’ illness and treatment experiences, with a view to understanding their potential influences on intervention acceptability. To our knowledge, it is the first study seeking to understand how the perspectives of primary care CWP patients may underpin treatment uptake, engagement and delivery.

## Method

Trial groups consisted of i) telephone-based Cognitive Behavioural Therapy (T-CBT); ii) prescribed exercise (PE); iii) combined T-CBT & PE (T-CBT/PE) and iv) usual GP care (UC). Trial participants were recruited from 8 general practices in North-West England & Scotland. Trial inclusion criteria comprised of adults with i) an American College of Rheumatology (ACR) definition of CWP in fibromyalgia [18], ii) impaired physical function as assessed by the Chronic Pain Grade Questionnaire [19], iii) a GP consultation for pain within the last 12 months and iv) access to a landline or mobile phone for the purposes of intervention delivery. All participants had to meet the ACR criteria for CWP but were not required to undergo the tender point examination necessary to confirm a fibromyalgia diagnosis. Participants were excluded from the trial if their GP highlighted contraindications to participation in any of the proposed interventions.

All participants received usual GP care. Those in the T-CBT intervention also received an initial 1 h telephone assessment followed by seven weekly T-CBT sessions of between 30 and 45 min duration. Two follow-up therapy sessions occurred at 3 and 6 months post assessment. T-CBT participants received a self-management workbook which included agreed collaborative goals for the therapist and patient to work towards, activity diaries and a range of monitoring sheets. CBT was delivered by experienced and accredited therapists who received 5 days additional training incorporating contextual CWP information and telephone skills practice. Clinical supervision was provided to therapists on a fortnightly basis.

Participants assigned to the PE arm were prescribed an exercise programme consistent with the American College of Sport Medicine (ACSM) guidelines for improving cardiorespiratory fitness [20]. Participants were advised to attend a designated local leisure facility at least twice per week for 20–60mins duration, where personal trainers would be available to guide them through an individually tailored programme. The specific mode of exercise reflected the personal preference of the participant. Initial exercise intensity was low to moderate and gradually increased until patients are exercising at a level consistent with the ACSM guidelines. Participants were free to engage in other modes of exercise, such as strength and flexibility training, as they wished and were also advised to engage in ‘everyday’ activities designed to enhance cardio-respiratory fitness (e.g. brisk walking) on those days that they did not attend. Fitness instructors received 1 day of additional training focusing on exercise prescription for individuals with chronic widespread pain. Thereafter they were encouraged to view participants in the same way that they would view clients without chronic widespread pain. Participants randomised to the combined arm received both of the interventions detailed above, with two-way information exchange encouraged between the CBT and PE facilitators.

Participation in the qualitative study was voluntary and not a pre-requisite to trial participation. Participants were invited into the qualitative study after the trial’s primary end point (6-month post-randomization) to avoid any reporting bias arising from interview participation. The first 100 people recruited to the trial and returning 9-month follow-up data were invited to take part. Sampling was purposeful in that it targeted participants with direct experience of the trial interventions. Maximum variation in geographical location was achieved by sampling across trial recruitment regions and sites. There was no minimum eligibility criterion for treatment engagement; only participants who had formally withdrawn from the trial were ineligible to take part.

All invited participants who expressed an interest in the qualitative study (*n* = 46) were telephoned by a researcher (PB) and provided with additional information regarding study procedures.

Following the receipt of written informed consent, interviews were conducted using open-ended, inductive questioning organised around broad topics identified and piloted among the research team. These included patients’ physical and emotional reactions to pain, their rationalisation of chronic or unexplained symptoms, their treatment preferences and the perceived fit between the trial interventions and patient need. Due to the geographical spread of study participants, all interviews were conducted on the telephone via a scheduled call to participants’ homes. Interviews were conducted by a health services researcher with over 10 years’ experience of conducting qualitative interviews (PB). The interviewer was not known to participants, had no prior experience of CWP, and introduced herself as a non-clinical researcher independent from the trial team.

Interviews were digitally recorded and transcribed verbatim. No field notes were taken during the telephone interviews as priority was given to establishing rapport with participants. Each interview was reviewed immediately after completion to confirm that all salient information had been picked up by the digital recordings. Interview duration was flexible and determined by the research participant. Interviews ranged from 30 to 60 min; mean (SD) 50.3 (8.0) minutes.

Participants were sent copies of their transcripts for editing and correction purposes; no changes to transcripts were made and no repeat interviews were conducted. Questionnaires administered as part of the trial provided baseline demographic, psychological and clinical data for the sample.

Resulting data were analysed by the framework method [21]. Data were coded inductively by PB using the method of constant comparison [22]. Codes were initially developed within each intervention group and then collectively applied across all four trial arms. A provisional analytical framework was built up by grouping into codes into clusters and then into emergent themes. During the constant comparison of new data, the framework was amended to allow for the introduction of new codes, and/or the removal of provisional codes that became superfluous during the course of analysis. Data coding and development of the analytical framework was managed in Microsoft Word. Data interpretation was facilitated by entering the indexed data into Microsoft Excel. A matrix was constructed in which participant was represented as rows and codes were represented as columns to facilitate data interpretation within and across cases. Analysis occurred in parallel to data collection and sampling continued until no new themes or sub-themes emerged. Verification of the analysis was provided by two other members of the research team who independently reviewed excerpts of the coding framework and agreed data interpretations and themes. All researchers were blinded to the trial’s quantitative results. Due to the geographical spread of the sample, participants’ were not asked to review the final analytical framework or its associated data trail.

Study methods are reported according to COREQ guidelines. Within the text that follows, participants (*P*) have been assigned a code number rather than a name or pseudonym. Participant gender (M/F) and trial allocation (TCBT, PE, COMBINED, UC) are provided.

## Results

**Fig. 1:**
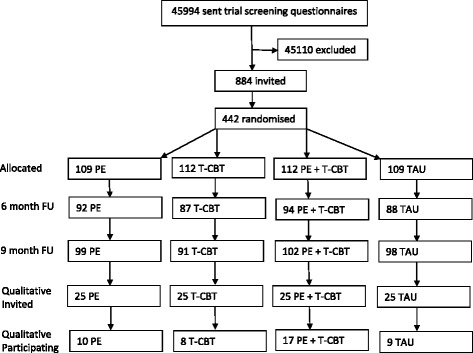
Participant flow

Participant flow through the trial and nested qualitative study is depicted in Fig. 1. Forty four participants contributed data to the qualitative analysis; 10 from the PE arm, 8 from T-CBT and 17 from the combined intervention. Nine participants from the usual care arm provided data relating to illness experiences, pain attributions and treatment needs. This nested sample was largely representative of the trial population (Table 1). Impaired physical function as assessed by the Chronic Pain Grade Questionnaire [20] ranged from grades 1–3; a slightly greater proportion of the interview sub-sample were classified at grade 1 or 2 reflecting a potentially higher pain severity to those participating in the main trial. Due to ethical restrictions, reasons for non-participation were not collated.

**Table 1:** Sample demographics for the nested qualitative study and trial samples

	Nested sample (*n* = 44)	Trial Sample (*n* = 442)
Mean (SD) age	58 (13)	56 (13)
% female	75	69
% fair-good health (self-report)	80	76
Mean (SD) GHQ score^a^	3.6 (3.7)	3.2 (3.6)
% CPG Grade 1–2^b^	56	69

^a^The GHQ (General Health Questionnaire) measures psychological distress from 0 (min) -12 (max). ^b^The CPG (Chronic Pain Grade questionnaire) classifies global pain severity from I [low disability, low intensity] to IV [high disability, severely limiting]

Three main themes emerged from the data. These themes were labelled: i) the illness context i.e. how people experience chronic pain & associated health services; ii) the identity context, referring to how people react to their symptoms and accommodate these within themselves and iii) the intervention context, denoting the extent and manner by which the different trial interventions aligned with these illness and identity responses.

### The illness context

The first theme, labelled here the illness context, comprised of three main sub-themes pertaining to participants’ symptom profiles, illness perceptions and help-seeking behaviours. Consistent with a CWP diagnosis, all participants reported acute pain episodes accompanied by chronic malaise.

*“It’s not consistent. You’ll have a while when it’s bad and then, whether you get used to it or not, I dunno, and then it just flares up again, it’s not a constant thing.” (41, F, COMBINED)*

Synonymous with the trial’s primary care recruitment strategies, all interviewees reported that they had consulted their family doctor to discuss their condition. A shared participant narrative highlighted weaknesses in this process, with common criticisms suggesting a lack of professional empathy and/or a failure to adequately explain or resolve the condition.

*“But you go to the doctor and it’s…well, you don’t seem to get any help at all. They never asked me how I was getting on or anything. There was just nothing, which I thought was quite amazing really!” (12, F, COMBINED)*

Patients emphasised a lack of personal understanding regarding their own pain triggers; a situation that they believed had been exacerbated by poor information provision and a lack of clinical consensus regarding the CWP experience. In the absence of a clear causal attribution for CWP, participants tended to conceive pain in physical terms, typically describing it as a natural warning system initiated in response to mechanical stress or dysfunction.

*“I mean you can go a week or two weeks and you’ve not got pain, and you just do something and you try to think, what did I do?.. You try to figure out, now what did I do? And you can’t think. How have I managed that? I mean I try, when I sit in a chair, I try to sit up as straight as I can, not lunge in the chair*…” (39, M, UC)

Participants’ help seeking behaviours were variable yet not without commonalities. Almost all had been referred for physiotherapy (from both statutory and private providers) and a notable number had also engaged in private osteopathy or acupuncture. Although considered effective in the short term, these interventions were often initiated in response to symptom exacerbation and accessed on an adhoc basis. Some participants perceived physiotherapy interventions to lack staff continuity. The vast majority attending physiotherapy or chiropractor appointments suggested that they were limited in terms of the amount of longer term relief that they could provide.

*“I mean I did go for, and I paid it myself, to a chiropractor, something like that but I didn’t find that helped- just that it was very expensive.”* (33, F, UC)

As a consequence most individuals relied on pharmacological intervention, whether GP or self-prescribed. Tension was evident between participants’ use of prophylactics to maintain roles and responsibilities, and patient perceptions of medication as a sub-optimal management strategy for chronic pain. Discourse suggested a tendency for patients to adjust recommended dosages downwards, a shared response that appeared to be driven by the fear of causing further structural damage when pain symptoms were artificially dulled.

*“Oh, I’ve had endless pills, things that confused me. I think everybody has problems with pain killers.” (41, F, COMBINED)*

### The identity context

The second emergent theme, the identity context, encompassed three main sub-themes pertaining to participants’ behavioural responses to pain, the interplay between participants self and illness identities and their emotional reactions to symptom progression.

Rarely did participants recognise any clear relationship between their own behaviour and the onset of pain. Only a small minority of patients reported pre-emptive adjustments to CWP, the deliberate use of activity pacing or practical lifestyle aids reflecting an unusual level of self-efficacy in the patient role. Many more described reactive responses.

*“What I packed into a week, you can’t keep going at that rate and not expect some fallout at the end of it, which normally was the bam or bust, I was either wham bam or I was absolutely knackered and bust.”* (40, F, COMBINED)

Three different storylines were identified in the data, each reflecting a different response to symptom exacerbation. The first, termed denial and distraction, described scenarios in which patients deliberately diverted their attention away from their pain in order to maintain their functional routines. The second, labelled ‘resistance’ described behaviour patterns that were consciously performed in order to challenge pain, while the third, named ‘tolerance,’ described attempts to sustain productivity by acknowledging and adjusting to pain:

*“Now to me I just say at times, mind over matter. I don’t have chronic pain. You can be in agony but you just try to shut off to it.”* (Denial & Distraction, 39, M, UC)

*“I’d be like, sod it, I’ll do it myself, I’ll manage, fine. It was if I permanently had to prove myself.” (*Resistance, 40, F, COMBINED)

*“I do bits, clear up a bit, then have a sit down. I usually get it done, just in a different way. I mean, it sometimes takes me longer and I’ve changed a bit what I do, but I still keep things clean and tidy, at least I hope I do!”* (Tolerance, 41 F, COMBINED).

Across all three storylines a shared narrative emerged on the outcomes of CWP, specifically the social and psychological impact of multiple role losses and a threat of self-deterioration. A disconnect emerged as participants strived to balance personal and social expectations of ‘a healthy self’ with a seemingly infinite need to accommodate CWP symptoms. Over time, and particularly during acute pain episodes, imposed role loss precipitated feelings of frustration, irritability or lowered mood:

*“I was very busy, I would go here there and everywhere, I was one of those people who looked after people, and then……I was going through a sort of process of having all sorts of things that seemed to be going wrong with me and restrictive movement and I was getting really quite depressed about it. I mean I’ve never had any treatment for depression but I was beginning to feel very down because you think “God, is this what it’s going to be like for the rest of my life?”* (12, F, COMBINED)

Key to understanding participant’s emotional reactions to pain was the observation that in almost all cases pain was experienced in cyclical episodes with no perceived control over their beginning or end. By implication, there was also no perceived control over the occurrence of future pain events. Sharing these experiences with others was difficult for some individuals, who ultimately feared that they would be regarded as lazy, or that the validity of their symptoms would be dismissed.

*“People can’t see it, and it’s hard to explain. Doing something one day and not the next, sometimes I’d worry, you know, that asking for help, well, that it sounds like an excuse…”* (40, F, COMBINED)

### The intervention context

The third and final theme, intervention context, comprised of two key sub-themes: Intervention preferences and intervention experiences. For clarity, data pertaining to this theme are presented separately for each trial intervention*.*

#### Acceptability of PE

As described previously, the MUSICIAN trial comprised four arms: i) telephone-based Cognitive Behavioural Therapy (T-CBT); ii) prescribed exercise (PE); iii) combined T-CBT & PE (T-CBT/PE) and iv) usual GP care (UC). All participants who were allocated to an intervention and who participated in post-intervention interviews (*n* = 35) expressed a pre-treatment preference for the PE arm. Qualitative data suggested that this preference was underpinned by three discrete but interacting biases.

Firstly, participant narratives revealed a strong experiential bias. The vast majority of the sample had accessed physiotherapy prior to the trial and a notable proportion had also seen osteopaths or chiropractors. Although arguably very different to a prescribed exercise intervention, these treatments were perceived to align logically with one another; each emphasising a need for physical manipulation. This viewpoint was in turn supported by a second bias, this time a conceptual bias that attributed pain to a structural weakness, something that was separate to the self and capable of repair:

“*It made some sense to me, yeah, I was an engineer, so that’s I see it you know. If it’s a problem with your joints, then that’s where you need a solution. Similar to physio in a way.. moving about, building up your muscles.. if you make the framework stronger, then yeah, that’s gonna help, I could see how that could work*.” (31, M, UC)

The final bias was a social bias, in which physical exercise was viewed as a positive health behaviour capable of delivering personal and population level benefits to health, productivity and appearance.

*“I mean I’m not lying in bed, I’ve never done that since I was in my twenties, you know, I just keep going, and I think exercise is going to be the answer.” (36, F, COMBINED)*

Participants who were allocated to, and engaged in, the exercise intervention highlighted multiple potential gains. Reports of symptom improvement were variable, but functional and social benefits were consistently reported. Participants valued meeting other individuals living with CWP at the gym and having the opportunity to normalise their pain experiences. Independently, prescribed exercise was perceived to renew or initiate interest in physical activity, reinforcing its perceived health value and increasing motivation for aerobic pursuits.

*“I wouldn’t have believed that I could actually do them but then I found I actually could, so my expectations were…it gave me more confidence because I could actually do it and also feel comfortable doing it. I was really quite pleased with myself because I thought, well, I have actually done it.”* (12, F, COMBINED)

Ultimately however, satisfaction with the exercise intervention varied widely. Of significance here was the recognition that many of the benefits that were identified by participants were considered long term gains. Critics of the PE intervention emphasised the substantial commitment that was required to initiate and sustain the programme. Negative emotional responses were evident among a small number of habitual non-exercisers who described an initial lack of enjoyment and self-efficacy for structured gym activities. Even among those who had previously exercised, motivation to engage in the intervention could be undermined by fears of functional loss.

*“It wasn’t fair to keep going to the gym and making myself -- because I was worse, so much worse when I’d been. So I thought, well,.. I’m not going to carry on doing it to make myself worse and suffer.”* (16, M, COMBINED)

With many individuals seeking to maintain occupational and social productivity, practical and logistical barriers to gym attendance were sometimes encountered. As such, the PE intervention was perceived to lack contextual relevance, mandating an increase in activity irrespective of fluctuating somatic symptoms or other demands on daily life.

*“It wasn’t easy and a couple of times, when I had lots on, I didn’t go. It seemed to take up a lot more time than you expected, getting there and changing. It did take up quite a lot of time. It needs a lot of planning really, because for me, well, I found it changes your routine.” (41, F, COMBINED)*

Participants looked directly to the facilitators of the exercise intervention (qualified gym instructors) to minimise their injury risk. Dissatisfaction with the programme occurred when instructors disengaged or when they were perceived to lack the appropriate personal and medical knowledge to deliver a safe, personalised intervention:

*“I told him I was having trouble with the bike, so he said right, okay, and the next time I went I realised that he’d written an extra lot of bikes on my sheet, like, woah! It didn’t make sense to me, somehow. After that, that’s when it all started to go downhill, really.”* (9, M, PE)

Regular facilitation with timely and appropriate responses to spontaneous pain events significantly elevated the acceptability of the PE intervention and emerged as a critical factor in patient engagement.

#### Acceptability of CBT

In direct contrast to the exercise intervention, cognitive behavioural therapy was often considered a less relevant and thus a less desirable intervention for chronic pain management. Lack of relevance was framed in different ways, but included at its core two key factors: a lack of fit with participants’ entrenched illness perceptions and a lack of fit with the self. Participants’ narratives revealed a lack of knowledge regarding the goals and remit of CBT and thus an initial lack of understanding regarding its ‘fit’ with a health condition predominantly attributed to physical causes:

*“And I was a bit -- I wasn’t flippant about it, I don’t mean in that way, but I was, like, I’m not quite sure what this is going to do for me, love, you’re really going to have to prove your point here.”* (40, F, COMBINED)

Shared discourse suggested that substantial stigma surrounded CBT use. At best, psychological therapy was perceived to question the validity of pain symptoms. At worst, it intimated that CWP was the result of an underlying character weakness requiring some sort of correction:

“Therapy, I mean, it’s one of those words, isn’t it. People think it’s for people who can’t cope, who aren’t strong enough to cope. I wasn’t sure what they were getting at I suppose, that I wasn’t in pain, that I had imagined that?” (39, M, UC)

Clear differences were observed between participants’ initial preferences for, and subsequent experiences of CBT. Several expressed relief at being able to share previously untold illness experiences. Many reported that direct interaction with a cognitive behavioural therapist had enabled them to benchmark their current daily routines and activity levels against social norms and identify potential self-care opportunities. Participants who had denied or challenged pain recounted how they had gradually begun to re-engage with their condition and legitimise their symptoms. Most believed that by engaging cognitive reflection they had been able to enhance their own understanding of pain triggers, thereby shifting the emphasis from reactive to proactive pain management strategies.

*“If I say to my husband, ‘I’m not going out, I really don’t want to,’ I don’t feel guilty now. Before I would never admit that, so from that perspective, things have changed, because I will say what I think and I will say no, I’m not doing it or I can’t do it. So the way I cope with my pain has changed.”* (40, F, COMBINED)

When asked, most participants expressed high satisfaction with an intervention delivered in situ. Telephone delivery was sometimes acknowledged to limit face to face interaction, limiting the depth of the relationship that could be established between a therapist and client. For the most part however, the impact of using a remote communication model was relatively minor. The key gains lay in its ability to overcome geographical or temporal access barriers, and to deliver timely and responsive behavioural change interventions into a contextually relevant setting.

*“It was fine, actually. And I think in some ways maybe it’s easier than face-to-face. I think if you had to make the appointment of somebody coming to the house it would be so much more difficult. And to be sat face-to-face with somebody might not necessarily be as easy as over the phone. Well, I suppose it’s like you, I can say anything, you don’t know whether I’m embarrassed or not. I can just say it..I can tell the truth, and it doesn’t matter.”* (11, F, TCBT)

Negative feedback regarding CBT for chronic pain focussed predominantly on the relevance of intervention resources. All participants allocated to CBT as part of the MUSICIAN trial were issued with a self-help manual. While diaries and written exercises were sometimes found to be useful, hypothetical case studies and lifestyle scenarios attracted criticism for their bias towards inactive and isolated individuals. Although the premise of providing patient case studies was rarely contested, the severity of the examples provided constituted an unnecessary and unwelcome reminder of potential identity loss for many. Engaging fully with therapy materials meant that participants had to be prepared to acknowledge this possibility and to perceive some relevance between the case studies and their own social and illness identities.

*“None of them, none of them related to anybody like me. The majority of people were people that were frightened of moving and frightened of doing things. They weren’t like me, I mean, I’ve got the opposite problem.” (36, F, COMBINED)*

#### Acceptability of combined PE and CBT

Although all data were combined for analysis, and thus contribute to the findings presented above, interviews with participants in the combined arm of the MUSICIAN trial (*n* = 17) provided a unique opportunity to explore the acceptability of a multi-component approach. Notably, no direct conflict between the physical and psychological components was reported. Rather, participant narratives highlighted the potential for the combined intervention to maximise the advantages and minimise the disadvantages of its two constituent components.

*“No, they’re two totally different things. On some occasions she’d say, oh, how are you today? And I was like: oh, I went to the bloody gym last night, and I know it today. But no, the two together for me were absolutely fine, they were totally different aspects, really, I had no problem having two of them.”* (40, F, COMBINED)

Whilst cognitive-behavioural intervention re-organised activity and lifestyle goals in order to make pain management more viable, prescribed exercise facilitated access to a structured mode of self-care promising improved physical function. Participants who received the combined intervention were less likely to conceptualise the CBT as a psychological therapy, and by implication, less likely to perceive stigma surrounding its use. Although the content and format of the CBT modality remained unchanged, participants were more inclined to conceive it as an informal resource, designed to support exercise uptake and maintenance. Multiple narratives upheld the conversational style of the psychological component, with some individuals explicitly regarding their therapist as a ‘coach’ or a ‘friend.’

*“You could tell her how things had worked in the gym and she could offer advice. It was helpful to have the two I would say have both if they got the chance. Yes I think that the talking did help. One goes along with the other, they work together.” (41, F, COMBINED)*

## Discussion

Chronic widespread pain (CWP) is associated with lost work productivity [5], mental ill health [6], and reduced quality of life [7]. Developing effective treatments is challenging. The present study sought to explore patient experiences of chronic widespread pain in an attempt to identify influences on treatment acceptability. Qualitative data were obtained from research trial participants allocated to a prescribed exercise programme and/or a psychological intervention (CBT).

There existed, at the start of the trial, a substantial tension between participants’ desires to maintain their daily routines and the extent to which pain symptoms and pain management strategies constrained these activities. Multiple role fulfilment was common in our sample. Although this could in part be justified by economic or domestic necessity, a shared patient narrative also emerged to confirm a social dimension to participants’ behaviour. Descriptions of habitual activities were typically framed in terms of their relevance to an individual, suggesting that substantial personal meaning was derived from their enactment. Lack of symptom visibility and casual attribution also heightened the risk that behavioural modification would be perceived negatively by others. Sociological theory, based on the Illness narratives of people living with other chronic conditions, confirms that physical impairment can intrude on a person’s life to the extent that it can undermine valued aspects of self-identity [23–25]. All modes of living are embedded in social context, and by implication, most individuals will take account of social attitudes when defining acceptable and non-acceptable behaviours. [23]. Our study thus suggests that health service providers must seek not only to understand people’s illness perceptions per se but the broader spheres of influence in which this illness is experienced.

Prior research has attended to the links between pain, the self and society [26–28] with specific attention directed towards the relationships between symptom disclosure, stigma and social exclusion [25, 29]. Successful adaptation to chronic illness is argued to depend upon an individual’s readiness to a) acknowledge their impairment and b) alter their life and self-identity in personally and socially acceptable ways [23, 28]. Varying levels of resistance were identified in our study, each tending towards a different level of symptom denial, dismissal or tolerance. Such behaviour is reminiscent of other categorisations of chronic illness responses, in which individuals are proposed to ignore, minimise or struggle against a burgeoning symptom profile [23]. Personal acceptance of CWP and its illness trajectory was rare among our sample, suggesting that effective self-management may not be an intuitive behaviour for primary care populations.

The novel contribution of our study lies in the exploration of patient perspectives as a key driver of uptake and engagement in internationally recommended CWP treatments [10]. Although intervention acceptability will inevitably be judged at the individual level, trends in our data suggest there may be some key differences between modes of intervention. Our study revealed that participants’ baseline preferences appeared to favour physical rather than psychological intervention. Qualitative analysis was undertaken blind to trial datasets; nevertheless these findings remain broadly consistent with quantitative data collected at the start of the MUSICIAN trial. Quantitative data reveal that 78 % of trial participants (*n* = 442) expressed a preference for physical exercise either alone (33 %) or in combination with CBT (45 %), 18 % expressed no preference and only 5 % preferred CBT as their sole intervention approach. Greater effort may thus be required to understand and overcome prejudices to psychological CWP intervention.

The parallels that have already been drawn between our data and the existing sociological literature provide one potential framework through which participants’ treatment preferences can be viewed. Referral to a prescribed exercise programme resonated most closely with participants’ tendency to attribute pain to a structural or mechanical defect. In doing so, it is also possible that it appealed sub-consciously or consciously, to their need to separate somatic symptoms from the self. Arguably, by objectifying pain and encouraging engagement in a socially-desirable health behaviour, the physical exercise intervention held considerable promise for personal and social identities threatened by ill-health. Psychological therapy in contrast, weakened this perspective. Among therapy-naïve individuals, CBT was perceived to denigrate pain, bringing with it connotations of social judgement, deviance and stigma. Potential differences in treatment meaning emphasise the importance of acknowledging different phases of illness acceptance and of providing relevant treatment options appropriate to each patient’s stage of reconciliation.

From a behavior change perspective, a logical rationale for a health intervention is rarely sufficient to ensure adequate uptake and participation [30–32]. A range of logistical, behavioral and psychosocial determinants of exercise behavior identified in the current study, many of which (e.g. competing lifestyle responsibilities and subjective perceptions of behavioural norms) resemble components of behavioural change theories developed for the general population [33]. Of particular interest to primary care CWP services however, may be the disproportionate influences that negative emotional reactions and/or a perceived lack of behavior control appear to exert on exercise participation. Our study participants tended towards concepts of CWP as a health state that could both affect, and be affected by, physical activity and functioning. In this sense, physical exercise was perceived as a high gain but high risk strategy, and fear of exacerbating pain symptoms was common. This finding resonates closely with previous work postulating relationships between pain-related fear, fear-avoidance and physical functioning in adults with chronic musculoskeletal pain [34]. Future applications of structured exercise programmes should acknowledge the context in which these interventions will be delivered, and ensure that all feasible opportunities to minimise risk and maximise motivation are taken. The need for exercise facilitators to be equipped with a minimum level of CWP knowledge and competency emerged as a critical element determining the acceptability of the prescribed exercise programme in the MUSICIAN trial, and has important implications for its resourcing and longer term integration.

Cognitive behavioural therapy, by definition does not pose equivalent physical risk. Our data suggest that the experiential gains of psychological therapy may ultimately exceed patient expectations. Through cognitive reflection and behavioural adaptation, participants may ultimately have experienced an upward identity shift independently of the need to increase physical exercise behaviour. Due consideration must nonetheless be given to the chosen delivery mechanism of any psychological therapies targeting chronic pain. Telephone delivery conferred substantial benefits for participants in the current study, both in terms of enhancing access and enabling ‘in situ’ intervention, delivering therapy directly into the context where behavioural change and/or pain management needed to occur. Among CWP populations, the use of a remote communication technology may ultimately help to overcome stigma and patient reticence for psychological therapy by reframing the therapeutic relationship as a non-clinical intervention providing socially-orientated support. Further study comparing patients’ views of the acceptability of different delivery models for CBT is recommended.

Our study suggests that the acceptability of primary care CWP interventions may be maximised when physical and psychological intervention are provided simultaneously, particularly where there is a pathway facilitating information exchange between the two. Nevertheless, uncertainty remains regarding the magnitude of acceptability gain, or the strength and nature of the relationship between treatment acceptability and effect. The MUSICAN trial^10^ has demonstrated that, when compared to treatment as usual (standard GP care), active intervention (PE, CBT and PE plus CBT) leads to clinically meaningful improvements in self-rated global health. Receiving the combined intervention (PE plus CBT) was associated with only a slightly better outcome than T-CBT alone and was considerably more expensive. Exploring patients’ treatment expectations at the point of assessment and referral, and ensuring that patients are informed and appropriately orientated to the premise and purpose of psychological intervention may ultimately be sufficient to ensure treatment uptake and engagement with a clinically and cost effective treatment.

This qualitative study has extended current understanding of CWP and pain management interventions by elucidating the likely acceptability of evidence-based treatments cited in interdisciplinary guidelines [11, 12]. Nesting a qualitative study within a randomised controlled raises the possibility of selection bias. Patients were initially recruited to the trial via self-report questionnaire, rather than during or immediately following primary care appointments. Assessing the accuracy of self-report consultation is challenging. Moreover, all interviewees had initially consented to participate in a randomised trial of CWP interventions and thus may be argued to display a level of openness towards the evaluated treatments atypical of a broader service population.

Trial process and outcome data were independently managed by a regulated clinical trials unit, prohibiting the early release of intervention outcome data. This ruled out the opportunity for maximum variation sampling based on treatment engagement or effect. Consent to participate in the nested qualitative study may have been motivated by a drive to report negative or positive treatment experiences.

Through our use of in-depth, semi-structured interviews, we allowed participants to raise issues that were important to them and which may not have arisen during a quantitative, questionnaire based study. All interviews were conducted on the telephone which reduced the researcher’s opportunity to draw on participants’ facial expressions and non-verbal cues. The impact of this on data depth and quality is not clear. The interviewer was experienced in conducting telephone interviews and in establishing telephone rapport. Telephone exchanges, when evaluated in the context of healthcare delivery, have been shown to overcome multiple access barriers including stigma [35, 36]. Non-face-to-face- communication can enhance a patient’s sense of anonymity, which may have particularly benefitted the current study given the potential impact of CWP on individuals’ self and social identities. When eligible participants are spread over a broad geographical area, telephone interviews can enhance recruitment and facilitate sampling across multiple recruitment sites. Due to access and ethical constraints, the views of participants who withdrew from the MUSCIAN trial, or who chose not to return their 9 month follow-up data, could not be explored.

## Conclusions

The acceptability of clinically recommended, evidence-based treatments for chronic widespread pain is influenced by a complex interplay of illness, social and self-identities. Patients’ pre-treatment preferences are likely to favour physical rather than psychological intervention but the experiential gains of psychological therapy typically exceed patient expectations. Potential differences in treatment meaning emphasise the importance of acknowledging different phases of illness acceptance and of providing the most appropriate treatment option for the stage of reconciliation. To maximise treatment adherence, health service providers should seek to understand individual perceptions of chronic widespread pain as well as the personal and social contexts in which this pain is experienced.
